# Global variation in grip strength: a systematic review and meta-analysis of normative data

**DOI:** 10.1093/ageing/afv192

**Published:** 2016-01-19

**Authors:** Richard M. Dodds, Holly E. Syddall, Rachel Cooper, Diana Kuh, Cyrus Cooper, Avan Aihie Sayer

**Affiliations:** 1 Medical Research Council Lifecourse Epidemiology Unit, University of Southampton, Southampton, UK; 2 Academic Geriatric Medicine, Faculty of Medicine, University of Southampton, Southampton, UK; 3 Medical Research Council Unit for Lifelong Health and Ageing at UCL, London, UK; 4 National Institute for Health Research Southampton Biomedical Research Centre, University of Southampton and University Hospital Southampton NHS Foundation Trust, Southampton, UK; 5 National Institute for Health Research Musculoskeletal Biomedical Research Unit, University of Oxford, Oxford, UK; 6 National Institute for Health Research Collaboration for Leadership in Applied Health Research and Care, Wessex, UK; 7 Newcastle University Institute for Ageing and Institute of Health and Society, Newcastle University, Newcastle, UK

**Keywords:** sarcopenia, grip strength, international, systematic review, old people

## Abstract

**Background:** weak grip strength is a key component of sarcopenia and is associated with subsequent disability and mortality. We have recently established life course normative data for grip strength in Great Britain, but it is unclear whether the cut points we derived for weak grip strength are suitable for use in other settings. Our objective was to investigate differences in grip strength by world region using our data as a reference standard.

**Methods:** we searched MEDLINE and EMBASE for reporting age- and gender-stratified normative data for grip strength. We extracted each item of normative data and converted it on to a *Z*-score scale relative to our British centiles. We performed meta-regression to pool the *Z*-scores and compare them by world region.

**Findings:** our search returned 806 abstracts. Sixty papers met inclusion criteria and reported on 63 different samples. Seven UN regions were represented, although most samples (*n* = 44) were based in developed regions. We extracted 726 normative data items relating to 96,537 grip strength observations. Normative data from developed regions were broadly similar to our British centiles, with a pooled *Z*-score 0.12 SDs (95% CI: 0.07, 0.17) above the corresponding British centiles. By comparison, normative data from developing regions were clearly lower, with a pooled *Z*-score of −0.85 SDs (95% CI: −0.94, −0.76).

**Interpretation:** our findings support the use of our British grip strength centiles and their associated cut points in consensus definitions for sarcopenia and frailty across developed regions, but highlight the need for different cut points in developing regions.

## Introduction

Weak grip strength is linked to a range of health outcomes including higher all-cause mortality rates [1–3] and morbidity [4, 5], as well as forming a key part of sarcopenia [6] and frailty [7] phenotypes. As such, there is growing interest in its assessment in clinical settings. We have recently established life course normative data for grip strength from 12 British studies, allowing an individual's strength to be assessed in terms of what would be expected for their gender and age [8]. Cut points for weak grip strength allow healthcare workers to easily identify older people who may benefit from further assessment: using a *T*-score approach with our normative data, we proposed cut points of 16 kg in females and 27 kg in males. These values are similar to those from the FNIH Sarcopenia Project (16 and 26 kg) based on the presence of mobility impairment in a sample combining several US and European cohorts [9]. This raises the question of whether our normative data are also applicable in US and European settings, as well as in other world regions. There exists a growing literature of normative data studies for grip strength covering different stages of the life course in different countries [10–15]. The objective of this paper was therefore to use a systematic review and meta-analysis to investigate differences in grip strength by world region, using our recently published life course normative data as a reference.

## Methods

### Literature search

We carried out a systematic review following the guidance in the PRISMA statement [16]. We searched the databases MEDLINE (including in-process citations) and EMBASE for English language publications up to August 2014 (for full search strategy, see Supplementary data, Appendix 2, available in *Age and Ageing* online). Eligible studies were those published from 1980 onwards reporting normative data for grip strength. We included studies based on samples of the general population and excluded those based on specific occupational or illness groups. We required studies to have reported their normative data in tabular form stratified by gender and age (across at most 15-year age bands) and to have included a mean and a standard error (or values from which one could be calculated) for each stratum.

### Data extraction

We extracted information about each study. In terms of the sample used, this included the country, level (national, regional, local or based in a single facility), the sample size and whether a sampling frame or a convenience sampling approach was used. We assumed a convenience sample had been used when the sample type was not described. In terms of the protocol used to measure grip strength, this included the dynamometer, measurement position (seated or standing), the hand(s) tested and the number of trials along with the summary value reported (mean or maximum).

For each age and gender stratum, we extracted the mean value for grip strength along with its standard error or equivalent values from which one could be calculated (the formulae used for these calculations are shown in Supplementary data, Appendix 3a, available in *Age and Ageing* online). We extracted the maximum value from both hands if this was reported; otherwise, we extracted the maximum or mean for the right or dominant side, as available in the paper. We converted grip strength values in pounds force and Newtons to kilograms force [17].

### Statistical analyses

We produced summary charts of the extracted normative data for males and females. As described in the results section, these data typically followed the same overall pattern across the life course as our British normative values. We therefore chose to use our British values as a reference, and converted the normative data items that we extracted from existing studies to mean *Z*-scores prior to further analyses. To do this, we calculated the difference between the mean from each normative data item and the mean from our British values for an equivalent age range, divided by the SD from our British values. Where a normative data item referred to a range of ages, we calculated the equivalent pooled mean and standard deviation [18] across the same age range in our British values and used these as the basis for *Z*-scores.

We used random-effects meta-regression in Stata version 13 [19] to pool the grip strength *Z*-scores and to investigate the associations with world region and aspects of measurement protocol. We assumed a coefficient of variation (the SD/mean) for grip strength of 0.25 (as seen in our British normative data) such that a difference of 0.4 on the *Z*-score scale is equivalent to a 10% difference in grip strength mean values (see Supplementary data, Appendix 3b, available in *Age and Ageing* online).We classified countries into those in developed and developing regions using groupings provided by the United Nations Statistics Division [20]. In terms of measurement protocol, we classified type of dynamometer as hydraulic (divided into the commonly used Jamar hydraulic dynamometer [21] or other hydraulic dynamometers), electronic or not specified. We also compared results from studies which had measured grip in the seated or standing positions, those which had reported values from one or both hands and those which showed the maximum or mean value from repeated trials.

## Results

### Study selection and characteristics

**Figure 1.**
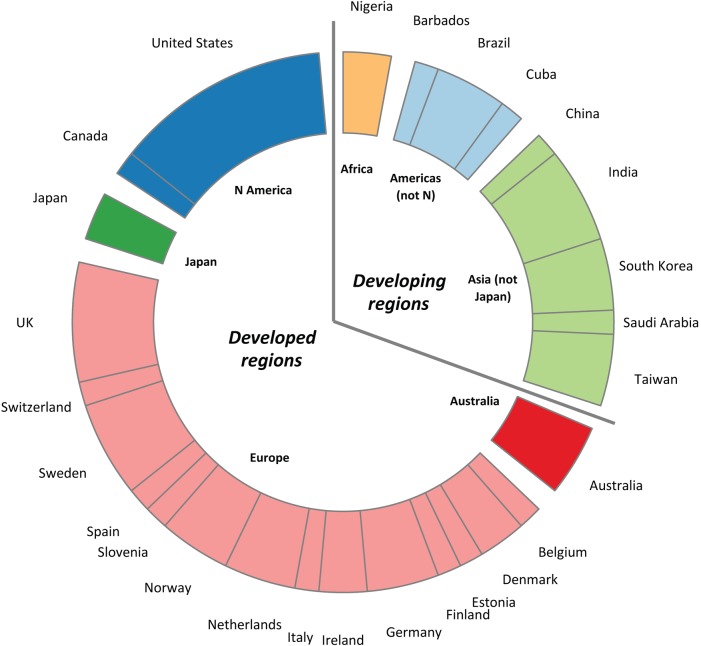
Country setting of included samples by UN region. The chart shows the country setting of the 63 included samples, grouped by UN region.

We screened 806 abstracts and assessed 96 papers for eligibility (for flow diagram, see Supplementary data, Appendix 4, available in *Age and Ageing* online). Sixty papers met inclusion criteria [10, 11, 13–15, 22–76]. Two papers included results for two samples [42, 62] and one paper provided results for two dynamometers [46]; hence the total number of samples for analysis was 63. A summary of their characteristics is given in Table 1, and a full list of included papers is provided in Supplementary data, Appendix 5, available in *Age and Ageing* online. The samples were based across 27 countries in seven UN regions, with a majority in developed regions (*n* = 44) as shown in Figure 1. The normative data covered childhood, adolescence and adulthood in four regions (Africa, Asia excluding Japan, Europe and Northern America). The data in the other three regions (Americas excluding Northern America, Australia and Japan) only covered adulthood. There were no samples from New Zealand (normally grouped with Australia in the UN classification).

**Table 1.** Characteristics of included samples, by developed status of region

Characteristic	Developed status, *n* (%)^a^		
	Developing (*N* = 19)	Developed (*N* = 44)	Both (*N* = 63)
Year of publication			
1985–94	2 (11)	6 (14)	8 (13)
1995–2004	2 (11)	15 (34)	17 (27)
2005–14	15 (79)	23 (52)	38 (60)
Sample level			
National	1 (5)	6 (14)	7 (11)
Regional	3 (16)	10 (23)	13 (21)
Local/facility/NS	15 (79)	28 (64)	43 (68)
Sample type			
Sampling frame	3 (16)	14 (32)	17 (27)
Convenience/NS	16 (84)	30 (68)	46 (73)
Sample size^b^			
Median (IQR)	435 (120, 1,005)	514 (270, 1,479)	498 (225, 1,119)
Stage of life course			
Child/adol. ≤18 years	3 (16)	11 (25)	14 (22)
Adults all <50 years	3^c^ (16)	2 (5)	5 (8)
Adults all ≥50 years	3 (16)	9 (20)	12 (19)
Adults, both ages	8 (42)	20^d^ (45)	28 (44)
All stages above	2 (11)	2 (5)	4 (6)
Dynamometer			
Jamar hydraulic	8 (42)	23 (52)	31 (49)
Other—hydraulic	6 (32)	12 (27)	18 (29)
Electronic	3 (16)	6 (14)	9 (14)
NS	2 (11)	3 (7)	5 (8)
Position			
Seated	10 (53)	32 (73)	42 (67)
Standing	6 (32)	7 (16)	13 (21)
NS	3 (16)	5 (11)	8 (13)
Hand(s) described in extracted data			
Right/dominant	18 (95)	32 (72)	50 (79)
Non-dominant	0 (0)	4 (9)	4 (6)
Both	1 (5)	8 (18)	9 (14)
Summary of trials			
Maximum	11 (58)	22 (50)	33 (52)
Mean	6 (32)	13 (30)	19 (30)
NS	2 (11)	9 (20)	11 (17)

NS, not specified.

^a^Unless otherwise specified. Please note all percentages are rounded to the nearest whole percentage point, and hence, the total for each group may not equal 100.

^b^This refers to the sample size for the age ranges extracted from each paper. This value is smaller than the sample size provided in papers which had included open-ended age ranges such as 75+ years.

^c^The paper by Chatterjee *et al.* [29] had an age range of 10–49 years, and for the purpose of this table, we classed this as a young adult paper.

^d^The paper by Backman *et al.* [24] had an age range of 17–70 years, and we classed this as adults, both ages.

### Relationship between world region and grip strength

**Figure 2.**
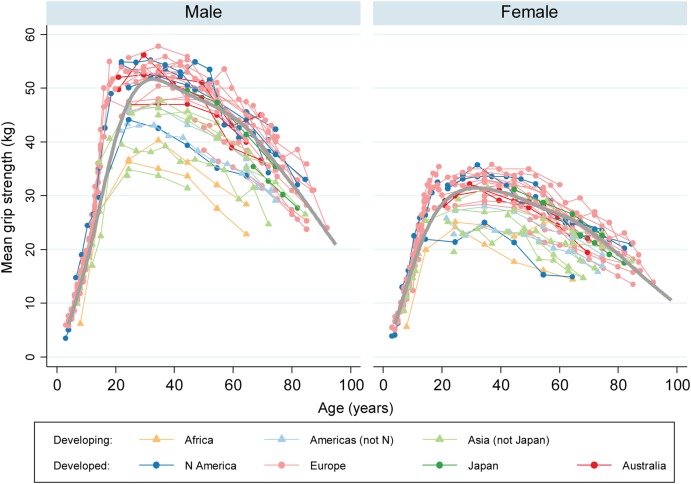
Grip strength mean values from included samples, by UN region. Each point represents the mean value of grip strength for each item of normative data, plotted against the mid-point of the age range it relates to. Values from the same sample are connected. Data from developing and developed regions are shown with triangles and circles, respectively. For comparison, the grey curve shows the mean values from our normative data for 12 British studies.

We extracted 726 normative data items relating to 96,537 grip strength observations. We excluded eight normative data items outside the age range of our British normative data (younger than 4 years or older than 90 years), leaving 718 for analyses. As shown in Figure 2, we saw a similar pattern of mean grip strength across the life course in both males and females to that from our British normative values: an increase to peak in early adult life, broad maintenance through to midlife and decline from midlife onwards. There was separation between data items from developed and developing regions. As shown in Table 2, Data items from developed regions were typically similar to those in Britain, with a pooled *Z*-score of 0.12 SDs (95% CI: 0.07, 0.17) above the equivalent British centiles. Data items from developing regions were typically lower, with a pooled *Z*-score of −0.85 SDs (95% CI: −0.94, −0.76). To illustrate these values, at age 30 in British males, we previously found mean grip strength to be 51.6 (9.6) kg. At this age, the pooled *Z*-scores from developed and developing regions would equate to mean grip strengths of 52.8 and 43.4 kg, respectively. The pooled results within each of the seven UN regions were consistent with this pattern although the number of samples contributing to three regions (Africa, Australia and Japan) was low.

**Table 2.** Pooled *Z*-scores by region status and individual regions

Classification	*N*^a^	Pooled *Z*-score^b^	(95% CI)	Adjusted *R*^2c^
Overall	63	−0.09	(−0.14, −0.04)	–
UN region status				
Developing	19	−0.85	(−0.94, −0.76)	34.1%
Developed	44	0.12	(0.07, 0.17)	
UN world region (with references shown)				
Developing regions				36.3%
Africa [13, 25]	2	−1.34	(−1.57, −1.11)	
Americas excluding N America [28, 62, 63, 71]	5	−0.80	(−0.97, −0.63)	
Asia excluding Japan [11, 23, 29, 30, 42, 67, 69, 72, 74, 75]	12	−0.74	(−0.86, −0.62)	
Developed regions				
Australia [46, 47]	3	−0.01	(−0.20, 0.18)	
Europe [10, 14, 15, 22, 24, 31–33, 35, 37, 38, 43–45, 50, 51, 53–61, 66, 68, 70, 73]	29	0.13	(0.07, 0.19)	
Japan [64, 76]	2	−0.13	(−0.40, 0.14)	
Northern America [26, 27, 34, 36, 39–41, 48, 49, 65]	10	0.16	(0.04, 0.28)	

Results shown are from separate meta-regression models of all 718 normative data items, with model term(s) those for each classification shown.

^a^*N*, number of samples contributing to each subgroup.

^b^The *Z*-score scale is the number of SDs above the equivalent values from our British centiles. Each pooled *Z*-score (and 95% CI) is from a meta-regression model combining the *Z*-scores for all the normative data items from the subgroup shown.

^c^The adjusted *R*^2^ is the proportion of variance between each item of normative data explained by each of the two classifications.

### Measurement protocol and reporting of normative data

The protocol used to measure grip strength and the reporting of normative data varied between the included papers. The majority had used the Jamar hydraulic (*n* = 31) or other types of hydraulic dynamometer (*n* = 18), with measurement in the seated position (*n* = 42). Most studies (*n* = 54) presented grip strength normative data for each hand separately (and we extracted data for either the right or dominant hand in such cases). The summary of repeated trials varied: the maximum (*n* = 33), mean (*n* = 19) or not specified (*n* = 11). Meta-regression analyses (results not shown) did not find evidence of a difference in mean grip strength *Z*-scores in terms of these protocol and reporting factors.

## Discussion

### Main findings

We carried out a systematic literature review of published normative data for grip strength. We saw that the normative data followed the same pattern across the life course as our previous British normative data, with an increase across childhood to peak in early adult life, broad maintenance through to midlife and decline from midlife onwards. There was clear evidence that average grip strength measurements are substantially lower in developing compared with developed world regions. Our findings are important since they highlight how consensus definitions of sarcopenia and frailty may need different cut points for grip strength for different geographical regions.

### Comparison with other studies

There are several possible explanations for the difference in normative data for grip strength between regions, including differences in body size and composition including mean height and weight. We were not able to test this as the studies did not present age- and gender-stratified height and weights alongside those for grip strength. Koopman *et al.* [77] compared grip strength between samples in Ghana and the Netherlands across ages 50–80 years: those in the Netherlands were stronger on average than those in Ghana, but they were also taller and had higher BMI. To take account of these differences in body size, the authors used linear regression to predict age- and gender-specific grip strength in the Ghanaian sample assuming they had the same mean height and BMI as their Dutch counterparts. This showed that differences in body size largely explained the differences in grip strength that had been observed. Clearly these differences in height and weight between Ghana and the Netherlands are likely to be explained by a wide range of factors including early growth, nutrition and genetic factors, many of which may also account for the differences seen in grip strength.

We are not aware of other studies that have compared normative data for grip strength between world regions. Two of the papers from our literature search provided normative values from two samples: Kaur *et al.* [42] from rural and urban Haryana in North India, and Rodrigues-Barbosa *et al.* [62] from Barbados and Cuba. In neither paper had the authors tested for differences between the two samples. We found no evidence of a marked or statistically significant difference when we did this by pooling the *Z*-scores from each sample (results not shown). Bohannon *et al.* [12] combined normative data for grip strength from 12 studies from countries in developed regions: the USA, Canada, UK, Sweden and Australia in order to produce normative data for the Jamar dynamometer for ages 20–75. They did not find evidence of heterogeneity between the 12 studies that they combined. This is in keeping with our results of similar pooled *Z*-scores from developed regions.

We are also not aware of other studies that have compared sets of normative data for grip strength collected with different measurement protocols. We previously found that 12 British studies using a range of dynamometers in the seated and standing positions produced acceptably similar normative data [8], and our present finding of no marked difference in the pooled *Z*-scores of other normative data papers by protocol factors is consistent with this. Several studies have examined whether values from repeat measurements of grip strength using different dynamometers [78–80] or a change of position [81–83] are consistent. The findings have varied, although overall these studies support using a consistent protocol for repeat measurements of an individual's grip strength where possible. We also again highlight the importance of recent calls for standardisation in future data collections [21, 84].

### Clinical relevance of findings

As consensus definitions for sarcopenia and frailty are implemented in clinical practice, the question of whether a single set of normative data and cut points for grip strength can be applied across a range of different countries is an important one. For example, we previously used a *T*-score approach [85] to produce cut points from our British normative data of 27 kg in males and 16 kg in females, and we estimated that ∼25% of individuals aged 80 were at or below these levels [8]. If the same cut-offs were applied to a hypothetical population with a mean grip strength of 0.85 SDs lower than our British norms, as seen in our pooled results for developing regions, then the prevalence at age 80 would increase to ∼60%. This suggests that the cut points from our British normative data may not be specific enough in developing regions. Indeed, the potential need for specific cut points for the Asia region, as well as the need to consider heterogeneity between countries in this region, has recently been recognised [86, 87]; a situation analogous to the use of region-specific BMD thresholds for fracture risk prediction in osteoporosis. It would be helpful to investigate prospective associations between grip strength and outcomes in a region-specific fashion [2], in order to determine optimum cut points for use in clinical practice.

A related question is whether normative data and cut points need to be stratified by height and ethnicity, and whether these factors would explain the regional differences that we observed. As described earlier, differences in height (and BMI) appeared to account for differences in grip strength between samples from Ghana and the Netherlands [77]. Other studies in the USA and the Netherlands have examined differences in grip strength by ethnicity [88–90]. The combination of data from cohorts based in different regions would allow the independent contributions of height, ethnicity and region to the global variation in grip strength to be examined.

We searched for papers containing normative data on grip strength from samples of the general population. The results of this review demonstrate that the measurement of grip strength has been undertaken in such samples in many countries, suggesting that it is acceptable for research participants and data collection teams. There is growing interest in the assessment of grip strength of individuals in hospitals and care homes [91–95] although to date there are few examples of age- and gender-stratified normative data in this diverse range of settings.

### Strengths and limitations

The systematic literature review had some limitations. In terms of the literature search, it is possible that there are other examples of normative data for grip strength in journals not indexed by MEDLINE or EMBASE, or those not published in medical journals, such as in government reports. We also found 12 papers with data not presented in the correct form. It is possible that contacting the authors of these papers would have allowed us to include them. However, we do not believe that the inclusion of additional papers would substantially alter our results. Also many of the included papers were based on small convenience samples of the local area or one facility. This may have led to pooled estimates for some countries which were not representative of the population as a whole although we did use random-effects meta-regression which anticipated variance between estimates and weighted them according to their standard errors. Finally as stated above, we have not been able to explore to what extent factors such as height and ethnic group account for the differences in grip strength between regions.

This review also had many strengths. We undertook a comprehensive literature search that yielded papers on grip strength from all world regions. There was considerable variation in how papers reported their normative data, such as the age ranges and descriptive statistics used. We undertook necessary data management and then used our British norms as a reference to generate *Z*-scores for inclusion in meta-regression analyses. As far as we are aware, such an approach has not been used before for grip strength.

### Conclusions

There is an urgent need for widely applicable thresholds for grip strength in men and in women. This systematic review found that normative data from developed regions were similar to those described in our recent British centiles, whereas those from developing regions were clearly lower. This supports the use of our cut points (or those from the FNIH Sarcopenia project) in consensus definitions for sarcopenia and frailty across Europe, Northern America, Australia and Japan. In Asia, the rest of the Americas and Africa, consideration will need to be given to region-specific cut points.

**Key points**

- Weak grip strength is a key component of sarcopenia and is associated with subsequent disability and mortality.
- We recently used data from British studies to develop cut points for grip strength of 16 and 27 kg in females and males, respectively.
- We examined global variation in grip strength and saw clear separation between mean values in developed and developing regions.
- Our findings suggest the need for region-specific cut points for grip strength in developing regions.

## Supplementary data

Supplementary data mentioned in the text are available to subscribers in *Age and Ageing* online.

## Conflicts of interest

None declared.

## Funding

This work was supported by the Wellcome Trust (Fellowship to R.M.D., grant number WT099055AIA); and the UK Medical Research Council (D.K. and R.C., programme code MC_UU_12019/4). The funders had no role in the design, execution, analysis and interpretation of data, or writing of the study.

## Supplementary Material

Supplementary Data
