# Supplementary material for: Global variation in grip strength: a systematic review and meta-analysis of normative data
Source: Age Ageing. 2016 Jan 19;45(2):209–16. doi: 10.1093/ageing/afv192 (PMC4776623; doi:10.1093/ageing/afv192)
Supplement: Supplementary Data [file supp_45_2_209__index.html]

Global variation in grip strength: a systematic review and meta-analysis of normative data — Global variation in grip strength: a systematic review and meta-analysis of normative data — Supplementary Data 

# Global variation in grip strength: a systematic review and meta-analysis of normative data

## Supplementary Data

Supplementary Data

- Supplementary Data - Docx file
