# Supplementary material for: Global variation in grip strength: a systematic review and meta-analysis of normative data
Source: Age Ageing. 2016 Jan 19;45(2):209–16. doi: 10.1093/ageing/afv192 (PMC4776623; doi:10.1093/ageing/afv192)
Supplement: Supplementary Data [file supp_afv192_afv192supp.docx]

# Supplementary material

## Appendix 1. Full list of references

1. Sayer AA, Kirkwood TBL. Grip strength and mortality: a biomarker of ageing? Lancet. 2015;386(9990):226–7.

2. Leong DP, Teo KK, Rangarajan S, *et al.* Prognostic value of grip strength: findings from the Prospective Urban Rural Epidemiology (PURE) study. Lancet. 2015;386(9990):266–73.

3. Cooper R, Kuh D, Hardy R, Mortality Review Group. Objectively measured physical capability levels and mortality: systematic review and meta-analysis. BMJ. 2010;341:c4467.

4. Cooper R, Kuh D, Cooper C, *et al.* Objective measures of physical capability and subsequent health: a systematic review. Age Ageing. 2011;40(1):14–23.

5. Cheung C-L, Nguyen U-SDT, Au E, Tan KCB, Kung AWC. Association of handgrip strength with chronic diseases and multimorbidity. Age (Dordr). 2013;35(3):929–941.

6. Aihie Sayer A, Robinson SM, Patel HP, Shavlakadze T, Cooper C, Grounds MD. New horizons in the pathogenesis, diagnosis and management of sarcopenia. Age Ageing. 2013;42(2):145–50.

7. Fried LP, Tangen CM, Walston J, *et al.* Frailty in older adults: evidence for a phenotype. J Gerontol A Biol Sci Med Sci. 2001;56A(3):M146–M156.

8. Dodds RM, Syddall HE, Cooper R, *et al.* Grip strength across the life course: normative data from twelve British studies. PLoS One. 2014;9(12):e113637.

9. Alley DE, Shardell MD, Peters KW, *et al.* Grip strength cutpoints for the identification of clinically relevant weakness. J Gerontol A Biol Sci Med Sci. 2014;69(5):559–66.

10. Häger-Ross C, Rösblad B. Norms for grip strength in children aged 4-16 years. Acta Paediatr. 2002;91(6):617–25.

11. Almuzaini KS. Muscle function in Saudi children and adolescents: relationship to anthropometric characteristics during growth. Pediatr Exerc Sci. 2007;19(3):319–33.

12. Bohannon RW, Peolsson A, Massy-Westropp N, Desrosiers J, Bear-Lehman J. Reference values for adult grip strength measured with a Jamar dynamometer: a descriptive meta-analysis. Physiother. 2006;92(1):11–15.

13. Adedoyin RA, Ogundapo FA, Mbada CE, *et al.* Reference values for handgrip strength among healthy adults in Nigeria. Hong Kong Physiother J. 2009;27(1):21–29.

14. Frederiksen H, Hjelmborg J, Mortensen J, McGue M, Vaupel JW, Christensen K. Age trajectories of grip strength: cross-sectional and longitudinal data among 8,342 Danes aged 46 to 102. Ann Epidemiol. 2006;16(7):554–62.

15. Spruit MA, Sillen MJH, Groenen MTJ, Wouters EFM, Franssen FME. New normative values for handgrip strength: results from the UK Biobank. JAMDA. 2013;14(10):775.e5–11.

16. Moher D, Liberati A, Tetzlaff J, Altman DG, The PRISMA Group. Preferred reporting items for systematic reviews and meta-analyses: the PRISMA statement. Ann Intern Med. 2009;151(4):264–269.

17. Engineering Department University of Cambridge. Unit conversion tables. Available at: http://www3.eng.cam.ac.uk/DesignOffice/cad/proewild3/usascii/proe/promec/getstart/units/reference/units_conv.htm. Accessed February 26, 2015.

18. Burton DA. Composite standard deviations. Available at: http://www.burtonsys.com/climate/composite_standard_deviations.html. Accessed December 11, 2014.

19. StataCorp. Stata statistical software: release 13. 2014.

20. United Nations Statistics Division. Composition of macro geographical (continental) regions, geographical sub-regions, and selected economic and other groupings. 2013. Available at: http://unstats.un.org/unsd/methods/m49/m49regin.htm. Accessed December 9, 2014.

21. Roberts HC, Denison HJ, Martin HJ, *et al.* A review of the measurement of grip strength in clinical and epidemiological studies: towards a standardised approach. Age Ageing. 2011;40(4):423–9.

22. Aadahl M, Beyer N, Linneberg A, Thuesen BH, Jørgensen T. Grip strength and lower limb extension power in 19-72-year-old Danish men and women: the Health2006 study. BMJ Open. 2011;1(2):e000192.

23. Ahn R, Yoo C-I, Lee H, *et al.* Normative data for neuromuscular assessment of the hand-arm vibration syndrome and its retrospective applications in Korean male workers. Int Arch Occup Environ Health 2013;86(7):837–44.

24. Backman E, Johansson V, Hager B, Sjoblom P, Henriksson KG. Isometric muscle strength and muscular endurance in normal persons aged between 17 and 70 years. Scand J Rehab Med. 1995;27:109–117.

25. Balogun JA, Adenlola SA, Akinloye AA. Grip strength normative data for the Harpenden dynamometer. J Orthop Sport Phys Ther. 1991;14(4):155–60.

26. Bear-Lehman J, Kafko M, Mah L, Mosquera L, Reilly B. An exploratory look at hand strength and hand size among preschoolers. J Hand Ther. 15(4):340–6.

27. Brennan P, Bohannon R, Pescatello L, Marschke L, Hanson S, Murphy M. Grip strength norms for elderly women. Percept Mot Skills 2004;99:899–902.

28. Budziareck MB, Pureza Duarte RR, Barbosa-Silva MCG. Reference values and determinants for handgrip strength in healthy subjects. Clin Nutr. 2008;27(3):357–62.

29. Chatterjee S, Chowdhuri BJ. Comparison of grip strength and isometric endurance between the right and left hands of men and their relationship with age and other physical parameters. J Hum Ergol. 1991;20:41–50.

30. Chuang MC, You M, Cai D, Chen CC. Isometric muscle strength of Chinese young males in Taiwan. Ergonomics. 1997;40(5):576–90.

31. Cohen DD, Voss C, Taylor MJD, Stasinopoulos DM, Delextrat A, Sandercock GRH. Handgrip strength in English schoolchildren. Acta Paediatr. 2010;99(7):1065–72.

32. Corish CA, Kennedy NP. Anthropometric measurements from a cross-sectional survey of Irish free-living elderly subjects with smoothed centile curves. Br J Nutr. 2003;89(1):137–45.

33. De Smet L, Vercammen A. Grip strength in children. J Pedr Ortho B. 2001;10:352–4.

34. Desrosiers J, Bravo G, Hébert R, Dutil É. Normative data for grip strength of elderly men and women. Am J Occ Ther. 1995;49(7):637–44.

35. Gunther CM, Burger A, Rickert M, Crispin A, Schulz C. Grip strength in healthy caucasian adults: reference values. J Hand Surg Am. 2008;33(4):558–65.

36. Hanten WP, Chen W-Y, Austin AA, *et al.* Maximum grip strength in normal subjects from 20 to 64 years of age. J Hand Ther 1999;12(3):193–200.

37. Harkonen R, Piirtomaa M, Alaranta H. Grip strength and hand position of the dynamometer in 204 Finnish adults. J Hand Surg [Eur]. 1993;18B:129–32.

38. Holm I, Fredriksen P, Fosdahl M, Vøllestad N. A normative sample of isotonic and isokinetic muscle strength measurements in children 7 to 12 years of age. Acta Paediatr. 2008;97(5):602–7.

39. Horowitz BP, Tollin R, Cassidy G. Grip Strength : Collection of normative data with community dwelling elders. Phys Occ Ther Ger. 1997;15(1):53–64.

40. Jansen CWS, Niebuhr BR, Coussirat DJ, Hawthorne D, Moreno L, Phillip M. Hand force of men and women over 65 years of age as measured by maximum pinch and grip force. J Aging Phys Act. 2008;16(1):24–41.

41. Kallman D, Plato C, Tobin J. The role of muscle loss in the age-related decline of grip strength: cross-sectional and longitudinal perspectives. J Gerontol. 1990;45(3):M82–8.

42. Kaur M. Age-related changes in hand grip strength among rural and urban Haryanvi Jat females. Homo. 2009;60(5):441–50.

43. Kenny RA, Coen RF, Frewen J, Donoghue OA, Cronin H, Savva GM. Normative values of cognitive and physical function in older adults: findings from the Irish Longitudinal Study on Ageing. J Am Geriatr Soc. 2013;61 S2:S279–90.

44. Lang I, Busche P, Rakhimi N, Rawer R, Martin DD. Mechanography in childhood: references for grip force, multiple one-leg hopping force and whole body stiffness. J Musculoskelet Neuronal Interact. 2013;13(2):227–35.

45. Luna-Heredia E, Martín-Peña G, Ruiz-Galiana J. Handgrip dynamometry in healthy adults. Clin Nutr. 2005;24(2):250–8.

46. Massy-Westropp N, Rankin W, Ahern M, Krishan J, Hearn TC. Measuring grip strength in normal adults: reference ranges and a comparison of electronic and hydraulic instruments. J Hand Surg Am. 2004;29(3):514–9.

47. Massy-Westropp NM, Gill TK, Taylor AW, Bohannon RW, Hill CL. Hand grip strength: age and gender stratified normative data in a population-based study. BMC Res Notes. 2011;4(1):127.

48. Mathiowetz V, Kashman N, Volland G, Weber K, Dowe M, Rogers S. Grip and pinch strength: normative data for adults. Arch Phys Med Rehabil. 1985;66:69–74.

49. Mathiowetz V, Wiemer DM, Federman SM. Grip and pinch strength: norms for 6- to 19-year-olds. Am J Occ Ther. 1986;40(10):705–11.

50. Molenaar HMT, Selles RW, Zuidam JM, Willemsen SP, Stam HJ, Hovius SER. Growth diagrams for grip strength in children. Clin Orthop Relat Res. 2010;468(1):217–23.

51. Montalcini T, Migliaccio V, Yvelise F, *et al.* Reference values for handgrip strength in young people of both sexes. Endocrine. 2013;43:342–45.

52. Mullerpatan RP, Karnik G, John R. Grip and pinch strength: normative data for healthy Indian adults. Hand Ther. 2013;18(1):11–16.

53. Nevill AM, Holder RL. Modelling handgrip strength in the presence of confounding variables: results from the Allied Dunbar National Fitness Survey. Ergonomics. 2000;43(10):1547–58.

54. Nilsen T, Hermann M, Eriksen CS, Dagfinrud H, Mowinckel P, Kjeken I. Grip force and pinch grip in an adult population: reference values and factors associated with grip force. Scand J Occ Ther. 2012;19(3):288–96.

55. Pearl A, Robinson D, Hale A. Fitness in a U.K. screening sample - a comparison with the Canadian population. Meth Inf Med. 1993;32:203–5.

56. Peolsson A, Hedlund R, Oberg B. Intra- and inter-tester reliability and reference values for hand strength. J Rehab Med. 2001;33:36–41.

57. Peters MJH, van Nes SI, Vanhoutte EK, *et al.* Revised normative values for grip strength with the Jamar dynamometer. J Peripher Nerv Syst 2011;16(1):47–50.

58. Ploegmakers JJW, Hepping AM, Geertzen JHB, Bulstra SK, Stevens M. Grip strength is strongly associated with height, weight and gender in childhood: a cross sectional study of 2241 children and adolescents providing reference values. J Physiother. 2013;59(4):255–61.

59. Puh U. Age-related and sex-related differences in hand and pinch grip strength in adults. Int J Rehab Res. 2010;33:4–11.

60. Rauch F, Neu CM, Wassmer G, *et al.* Muscle analysis by measurement of maximal isometric grip force: new reference data and clinical applications in pediatrics. Pediatr Res. 2002;51(4):505–10.

61. Ribom EL, Mellström D, Ljunggren Ö, Karlsson MK. Population-based reference values of handgrip strength and functional tests of muscle strength and balance in men aged 70-80 years. Arch Gerontol Ger. 2011;53(2):e114–7.

62. Rodrigues-Barbosa A, Miranda LM De, Vieira-Guimaraes A, Xavier-Corseuil H, Weber-Corseuil M. Age and gender differences regarding physical performance in the elderly from Barbados and Cuba. Rev Salud Pública. 2011;13(1):54–66.

63. Schlüssel MM, dos Anjos LA, de Vasconcellos MTL, Kac G. Reference values of handgrip dynamometry of healthy adults: a population-based study. Clin Nutr. 2008;27(4):601–7.

64. Seino S, Shinkai S, Fujiwara Y, *et al.* Reference values and age and sex differences in physical performance measures for community-dwelling older Japanese: a pooled analysis of six cohort studies. PLoS One. 2014;9(6):e99487.

65. Sella GE. The hand grip: gender , dominance and age considerations. Eur Med Phys. 2001;37(3):161–170.

66. Semproli S, Brasili P, Toselli S, Ventrella A, Jurimae J, Jurimae T. The influence of anthropometric characteristics to the handgrip and pinch strength in 6-10-year old children. Anthr Anz. 2007;65(3):293–302.

67. Shim JH, Roh SY, Kim JS, *et al.* Normative measurements of grip and pinch strengths of 21st century Korean population. Arch Plast Surg. 2013;40(1):52–6.

68. Skelton DA, Greig CA, Davies JM, Young A. Strength, Power and related functional ability of healthy people aged 65–89 years. Age Ageing. 1994;23(5):371–377.

69. Tsang RCC. Reference values for 6-minute walk test and hand-grip strength in healthy Hong Kong Chinese adults. Hong Kong Physiother J. 2005;23(1):6–12.

70. Tveter AT, Dagfinrud H, Moseng T, Holm I. Health-related physical fitness measures: reference values and reference equations for use in clinical practice. Arch Phys Med Rehabil. 2014;95(7):1366–73.

71. Vianna LC, Oliveira R, Araujo CGS. Age-related decline in handgrip strength differs according to gender. J Strength Cond Res. 2007;21(4):1310–14.

72. Wang C-Y. Hand dominance and grip strength of older Asian adults. Percept Mot Skills. 2010;110:897–900.

73. Werle S, Goldhahn J, Drerup S, Simmen BR, Sprott H, Herren DB. Age- and gender-specific normative data of grip and pinch strength in a healthy adult Swiss population. J Hand Surg [Eur]. 2009;34(1):76–84.

74. Wu S-W, Wu S-F, Liang H-W, Wu Z-T, Huang S. Measuring factors affecting grip strength in a Taiwan Chinese population and a comparison with consolidated norms. Appl Ergon. 2009;40(4):811–5.

75. Yim SY, Cho JR, Lee IY. Normative data and developmental characteristics of hand function for elementary school children in Suwon area of Korea: grip, pinch and dexterity study. J Korean Med Sci. 2003;18(4):552–8.

76. Yoshimura N, Oka H, Muraki S, *et al.* Reference values for hand grip strength, muscle mass, walking time, and one-leg standing time as indices for locomotive syndrome and associated disability: the second survey of the ROAD study. J Orthopaed Sci. 2011;16(6):768–77.

77. Koopman JJE, van Bodegom D, van Heemst D, Westendorp RGJ. Handgrip strength, ageing and mortality in rural Africa. Age Ageing. 2015;44(3):465–470.

78. Svantesson U, Nordé M, Svensson S, Brodin E. A comparative study of the Jamar® and the Grippit® for measuring handgrip strength in clinical practice. Isokinet Exerc Sci. 2009;17:85–91.

79. Amaral JF, Mancini M, Novo Júnior JM. Comparison of three hand dynamometers in relation to the accuracy and precision of the measurements. Rev Bras Fisioter. 2012;16(3):216–24.

80. Guerra RS, Amaral TF. Comparison of hand dynamometers in elderly people. J Nutr Heal Ageing. 2009;13(10):907–12.

81. Shechtman O, Mackinnon L, Locklear C. Using the BTE Primus to measure grip and wrist flexion strength in physically active wheelchair users: an exploratory study. Am J Occup Ther. 2001;55(4):393–400.

82. Balogun J, Akomolafe C, Amusa L. Grip strength: effects of testing posture and elbow position. Arch Phys Med Rehabil. 1991;72(5):280–3.

83. Liao W-C, Wang C-H, Yu S-Y, Chen L-Y, Wang C-Y. Grip strength measurement in older adults: a comparison of three testing positions. Austr J Ageing. 2014;33(4):278–82.

84. Reuben DB, Magasi S, McCreath HE, *et al.* Motor assessment using the NIH Toolbox. Neurology. 2013;80:S65–75.

85. Bohannon RW, Magasi S. Identification of dynapenia in older adults through the use of grip strength *t*-scores. Muscle Nerve. 2015;51(1):102–105.

86. Woo J, Arai H, Ng TP, *et al.* Ethnic and geographic variations in muscle mass, muscle strength and physical performance measures. Eur Geriatr Med. 2014;5(3):155–164.

87. Chen LK, Liu LK, Woo J, *et al.* Sarcopenia in Asia: Consensus report of the Asian working group for sarcopenia. JAMDA. 2014;15(2):95–101.

88. Haas SA, Krueger PM, Rohlfsen L. Race / ethnic and nativity disparities in later life physical performance: the role of health and socioeconomic status over the life course. J Gerontol Ser B Psychol Sci Soc Sci. 2012;67:238–248.

89. Van der Kooi A-LLF, Snijder MB, Peters RJG, van Valkengoed IGM. The association of handgrip strength and type 2 diabetes mellitus in six ethnic groups: an analysis of the HELIUS study. PLoS One. 2015;10(9):e0137739.

90. Araujo AB, Chiu GR, Kupelian V, *et al.* Lean mass, muscle strength, and physical function in a diverse population of men: a population-based cross-sectional study. BMC Public Health. 2010;10:508.

91. Kerr A, Syddall HE, Cooper C, Turner GF, Briggs RS, Aihie Sayer A. Does admission grip strength predict length of stay in hospitalised older patients? Age Ageing. 2006;35(1):82–4.

92. García-Peña C, García-Fabela LC, Gutiérrez-Robledo LM, García-González JJ, Arango-Lopera VE, Pérez-Zepeda MU. Handgrip strength predicts functional decline at discharge in hospitalized male elderly: a hospital cohort study. PLoS One. 2013;8(7):e69849.

93. Guerra RS, Fonseca I, Pichel F, Restivo MT, Amaral TF. Handgrip strength and associated factors in hospitalized patients. J Parenter Enter Nutr. 2015;39(3):322–30.

94. Keevil VL, Mazzuin Razali R, Chin AV, Jameson K, Aihie Sayer A, Roberts H. Grip strength in a cohort of older medical inpatients in Malaysia: a pilot study to describe the range, determinants and association with length of hospital stay. Arch Gerontol Ger. 2013;56:155–159.

95. Roberts HC, Syddall HE, Sparkes J, *et al.* Grip strength and its determinants among older people in different healthcare settings. Age Ageing. 2014;43(2):241–6.

## Appendix 2. Search strategy used

Search run on 11^th^ August 2014 using databases MEDLINE (including in-process citations) and EMBASE.

| **Step** | **Search string** | **Abstracts returned** |
| --- | --- | --- |
| 1 | (Hand Strength/ or Muscle Strength Dynamometer/ or "grip strength".ti,ab or "hand strength".ti,ab or "handgrip strength".ti,ab or "grip dynamometer".ti,ab) | 26480 |
| 2 | Reference Values/ or "reference values".ti,ab. or "normative".ti,ab. or (association* adj2 age).ab. or (relationship adj2 age).ab. or "age related".ti. or "age-related".ti. or "normal values".ti,ab | 334730 |
| 3 | 1 and 2 | 1167 |
| 4 | remove duplicates from 3  (with preference towards MEDLINE) | 860 |
| 5 | (4 and humans/) or (4 not (humans/ or animals/)) | 840 |
| 6 | limit 5 to english language | 811 |
| 7 | limit 6 to yr="1980 -Current" | 806 |

## Appendix 3. Statistical appendix

**a. Formulae used to calculate standard errors**

Calculation of standard error of the sample mean, $se$ from standard deviation, $s$ and sample size, $n$:

$$se=\frac{s}{\sqrt{n}}$$

Calculation of standard deviation, $s$, when alternatives provided as shown:

| Lower 95 % CI, ${CI}_{L}$ for mean $\bar{x}$ | $s=\frac{\left( \bar{x}-{CI}_{L} \right)*\sqrt{n}}{1.96}$ |
| --- | --- |
| Interquartile range^*^ $P_{25}$ and $P_{75}$ (based on the N(0,1) distribution, $P_{25}$ and $P_{75}$ are 0.674 SDs either side of the mean, so the difference between them represents 1.348 SDs). | $s=\frac{P_{75}-P_{25}}{1.348}$ |
| Fifth centile $P_{5}$  (as above, $P_{5}$ is 1.645 SDs below the mean) | $s=\frac{\left( \bar{x}-P_{5} \right)}{1.645}$ |

^*^ From our earlier work on normative data, we considered it reasonable to assume that grip strength was normally distributed and indeed on inspection of the data that we extracted from studies reporting a median and interquartile range for grip strength, there was very little evidence of skew.

**b. Interpretation of differences in grip strength on the Z-score scale**

In our paper on British normative data for grip strength, we tested if the results from sensitivity analyses were acceptably similar to our main grip strength centiles using a range of 10% either side of the main findings [**8**]. We took a similar approach in the current work when testing differences between our British normative data and the results of our systematic literature search, both in terms of world region and aspects of measurement protocol.

To do this, we assumed that the coefficient of variation (the ratio of the standard deviation to the mean) for grip strength was 0.25. This was based on our British normative data, where we saw a mean coefficient of variation across the life course of 0.22. The average coefficient of variation across the 730 normative data items in the present study was similar at 0.21.

Following our assumption, a 1 SD difference in mean grip strength is equivalent to a 25% difference in mean grip strength (kg). It therefore follows that a 10% difference in mean grip strength (kg) is equivalent a 0.4 SD difference in mean grip strength Z-score.

## Appendix 4. Flow diagram for systematic review

**n = 806 abstracts screened**

**n = 96 papers retrieved for further assessment**

**n = 60 papers included in the review**

n = 702 not considered relevant

n = 3 seen to be duplicates

n = 4 conference abstracts (no paper found)

n = 709 abstracts excluded:

n = 18 not a normative data study of grip strength

n = 36 papers excluded:

n = 4 specific illness / occupational groups

n = 12 normative data not in correct form*

n = 1 potentially relevant paper not available

n = 2 same data as an earlier study

* Reasons for normative data not being in the correct form included not presenting data in the form of a table, including a mean but no measure of spread such as standard deviation, and using an age range for each item of normative data wider than 15 years.

## Appendix 5. Full list of included studies

Studies ordered by first author and then by year. C, convenience sample. NS, not specified. Sf, sampling frame used.

^*^ The age range shown is that for the normative data items that we extracted from each paper. We excluded open-ended age ranges, such as 70+ years.

^†^ N, the number of individuals with grip strength measurements in the age range specified. These are notably lower than the figures in the published papers by Frederiksen et al. and Spruit et al., since in these two studies we chose to extract details for a single height group only.

^‡^ In the paper by Frederiksen et al., sample sizes for individual normative data items were not provided. Rather the sample size shown is approximated based on the standard deviation and standard error of the sample mean, which were provided for each item.

| **Author, year**  **Ref** | **Type** | **Country** | **Level** | **Sample description** | **Dynamometer** | **Age range (y)^*^** | **N^†^** |
| --- | --- | --- | --- | --- | --- | --- | --- |
| Aadahl, 2011  [22] | Sf | Denmark | Regional | Participants recruited through the Danish Civil Registration office across in 11 municipalities in the western area of the Capital Region of Denmark. | Jamar hydraulic | 19 - 72 | 3453 |
| Adedoyin, 2009  [**13**] | C | Nigeria | Facility | Participants recruited by advertisement and invitations from Obafemi Awolowo University, Ile-Ife. | Takei TKK 84466 | 20 - 69 | 745 |
| Ahn, 2013  [23] | C | Korea, Republic of | Facility | Recruited from healthy participants visiting the Occupational and Environmental Health Center over a 2 month period. | Camry Electronic | 30 - 59 | 120 |
| Almuzaini, 2007  [**11**] | C | Saudi Arabia | Local | Drawn from three local schools. | NS | 11 - 19 | 44 |
| Backman, 1995  [24] | Sf | Sweden | NS | Two sources mentioned in paper; appears approached all healthy adults in a small area of Linköping. | Rank Stanley Cox strain gauge | 17 - 70 | 128 |
| Balogun, 1991  [25] | C | Nigeria | Local | Participants were recruited from community residential quarters, shopping centers, churches, and schools. | Harpenden | 7 - 69 | 840 |
| Bear-Lehman, 2002  [26] | C | United States | Local | From 4 preschools in New York City. | Jamar hydraulic | 3 - 5 | 81 |
| Brennan, 2004  [27] | C | United States | Local | Noninstitutionalised women who participated in health screenings at one of five community senior centers in the state of Connecticut. | Jamar hydraulic | 60 - 89 | 104 |
| Budziareck, 2008  [28] | C | Brazil | Local | Three locations: a hospital, centre for older people and a local city square. | Jamar hydraulic | 18 - 30 | 100 |
| Chatterjee, 1991  [29] | NS | India | NS | Normal healthy male subjects. | Simple handgrip dynamometer -INCO made in India | 10 - 49 | 81 |
| Chuang, 1997  [30] | C | Taiwan, Province of China | Facility | From one junior college; participants paid NT$ 50 (approx 2 US $) for every hour attending session. | Takei TKK Muscular Power Measuring Device with digital dynamometer | 16 - 20 | 120 |
| Cohen, 2010  [31] | Sf | United Kingdom | Regional | 23 state primary and secondary schools in East of England Healthy Hearts study. | Takei T.K.K.5001 Grip A | 10 - 15 | 6683 |
| Corish, 2003  [32] | C | Ireland | Local | Recruited from interest groups for the active retired. | Takei | 65 - 85 | 874 |
| De Smet, 2001  [33] | NS | Belgium | NS | No sample details provided. | Jamar hydraulic | 5 - 15 | 419 |
| Desrosiers, 1995  [34] | Sf | Canada | Local | Random sampling (with replacement) from the electoral list | Jamar hydraulic | 60 - 79 | 240 |
| Frederiksen, 2006  [**14**] | Sf | Denmark | National | Participants of three nationwide population-based surveys. | Smedley | 45 - 94 | 2926^‡^ |
| Gunther, 2008  [35] | C | Germany | Regional | Volunteers randomly chosen from different locations including hospitals, public recreations areas and homes for the elderly. | Baseline digital hydraulic dynamometer | 20 - 95 | 769 |
| Hager-Ross, 2002  [**10**] | C | Sweden | Local | From 20 randomly chosen day care centres and schools in the municipality of Umea. | Grippit | 4 - 16 | 530 |
| Hanten, 1999  [36] | C | United States | NS | NS. | Jamar hydraulic | 20 - 64 | 1182 |
| Harkonen, 1993  [37] | NS | Finland | NS | Volunteers working in the food and medicine industries. | Jamar hydraulic | 30 - 49 | 115 |
| Holm, 2008  [38] | C | Norway | Local | Schools in the Oslo area up to 4-5km from hospital where study based. | Jamar hydraulic | 7 - 29 | 376 |
| Horowitz, 1997  [39] | C | United States | Local | Two Suffolk County, Long Island senior citizen community organisations. | Jamar hydraulic | 70 - 74 | 47 |
| Jansen, 2008  [40] | C | United States | Local | Recruited from local health fairs, a geriatric primary-care clinic and senior-citizen community events. | Jamar hydraulic | 65 - 84 | 196 |
| Kallman, 1990  [41] | Sf | United States | Regional | Baltimore Longitudinal Study of Ageing | Smedley | 20 - 89 | 842 |
| Kaur (rural), 2009  [42] | NS | India | Regional | Samples of rural Jat (the most prominent caste) females from Haryana, North India. | NS | 40 - 70 | 300 |
| Kaur (urban), 2009  [42] | NS | India | Regional | Samples of urban Jat (the most prominent caste) females from Haryana, North India. | NS | 40 - 70 | 300 |
| Kenny, 2013  [43] | Sf | Ireland | National | Nationally representative sample of adults. | Baseline | 50 - 85 | 5819 |
| Lang, 2013  [44] | NS | Germany | Facility | All participants from Tuebingen Waldorf School | Jamar hydraulic | 3 - 19 | 869 |
| Luna-Heredia, 2005  [45] | C | Spain | Local | Workers of the Móstoles Hospital, Madrid, relatives of patients visiting the hospital and elderly subjects from senior residences in two cities near Madrid. | Baseline and Grip-D (two devices used, considered exchangeable) | 30 - 84 | 473 |
| Massy-Westropp (Grippit), 2004  [46] | C | Australia | Local | From several sources including a large teaching hospital, a high pedestrian-traffic area of a Medical Centre and community centres. | Grippit | 18 - 74 | 362 |
| Massy-Westropp (Jamar), 2004  [46] | C | Australia | Local | From several sources including a large teaching hospital, a high pedestrian-traffic area of a Medical Centre and community centres. | Jamar hydraulic | 18 - 74 | 359 |
| Massy-Westropp, 2011  [47] | Sf | Australia | Regional | Data obtained from the North West Adelaide Health Study - random sampling using telephone directory. NB Sample size not divided into males and females, so total divided by two and split equally across age groups. | Jamar hydraulic | 20 - 69 | 2629 |
| Mathiowetz, 1985  [48] | C | United States | Regional | Recruited from shopping centers, fairs, senior citizen centers, a rehabiliation center (staff) and a university. | Jamar hydraulic | 20 - 74 | 577 |
| Mathiowetz, 1986  [49] | C | United States | Regional | Participants from schools in the seven-county Milwaukee area. | Jamar hydraulic | 6 - 19 | 471 |
| Molenaar, 2010  [50] | C | Netherlands | Facility | Children from a local primary school. | Lode | 4 - 12 | 225 |
| Montalcini, 2013  [51] | C | Italy | Facility | Healthy university students. | Hersteller | 19 - 25 | 335 |
| Mullerpatan, 2013  [52] | C | India | Facility | Students and staff members of (presumed) a single hospital. | Jamar hydraulic | 18 – 30 | 1005 |
| Nevill, 2000  [53] | Sf | United Kingdom | National | Random sample of English population with subsample having physical appraisal. | Nottingham electronic | 16 - 74 | 2632 |
| Nilsen, 2012  [54] | C | Norway | Local | Several settings including shopping malls, workplaces and community centres for the elderly in the region of Oslo. | Grippit | 20 - 79 | 498 |
| Pearl, 1993  [55] | C | United Kingdom | Facility | Subject attending BUPA Health Screening Centre, London. Of those over 50, only those who exercised regularly completed grip strength assessment. | NS | 20 - 69 | 16980 |
| Peolsson, 2001  [56] | Sf | Sweden | Facility | Age stratified sample of hospital staff. | Jamar hydraulic | 25 - 65 | 101 |
| Peters, 2011  [57] | C | Netherlands | Local | University, hospital and secondary school personnel, homes for the elderly and sports clubs. | Jamar hydraulic | 20 - 79 | 614 |
| Ploegmakers, 2013  [58] | C | Netherlands | Regional | Schools approached in the four northern provinces of The Netherlands. | Jamar hydraulic | 4 - 14 | 2241 |
| Puh, 2010  [59] | C | Slovenia | NS | Recruited at locations including shopping centres, fairs and nursing homes. | Baseline | 20 - 79 | 199 |
| Rauch, 2002  [60] | NS | Germany | Regional | Participants in the Dortmund Nutritional and Anthropometric Longitudinally Designed study. | Jamar hydraulic | 7 - 18 | 305 |
| Ribom, 2011  [61] | Sf | Sweden | Regional | MrOS (osteoporotic fractures in men) Sweden cohort in Uppsala. | Jamar hydraulic | 70 - 75 | 548 |
| Rodrigues-Barbosa (Barbados), 2011  [62] | Sf | Barbados | Local | Data taken from SABE (Survey on Health, Aging and Well Being in Latin America and the Caribbean), specifically Bridgetown. | Takei TK 1201 | 60 - 79 | 1119 |
| Rodrigues-Barbosa (Cuba), 2011  [62] | Sf | Cuba | Local | Data taken from SABE (Survey on Health, Aging and Well Being in Latin America and the Caribbean), specifically Havana. | Takei TK 1201 | 60 - 79 | 1425 |
| Schlussel, 2008  [63] | Sf | Brazil | Local | Three stage sampling procedure in the city of Niterói. | Jamar hydraulic | 20 - 69 | 2802 |
| Seino, 2014  [64] | Sf | Japan | National | Six cohort studies participating in TMIG-LISA (Tokyo Metropolitan Institute of Gerontology-Longitudinal Interdisciplinary Study on Aging). | Smedley-like | 65 - 84 | 4443 |
| Sella, 2001  [65] | C | United States | Facility | Retrospective analysis of data collected from an occupational physician's patients (none had upper limb pathology). | Jamar hydraulic | 10 - 69 | 860 |
| Semproli, 2007  [66] | C | Estonia | Local | Several schools in Tartu. | Takei TKK 5001 | 6 - 10 | 461 |
| Shim, 2013  [67] | C | Korea, Republic of | Facility | Patients visiting a hospital for normal health screening visits. | Jamar hydraulic | 10 - 79 | 336 |
| Skelton, 1994  [68] | C | United Kingdom | Local | Volunteers recruited through local and national newspapers to attend Human Performance Laboratory in Hampstead, London. | Takei Kiki Kogyo Handgrip mechanical dynamometer | 65 - 89 | 100 |
| Spruit, 2013  [**15**] | C | United Kingdom | National | Recruitment via centrally  coordinated identification and invitation from population-based registers (such as those held by the NHS) of potentially eligible people living within a reasonable travelling distance of an assessment centre. | Jamar hydraulic | 45 - 64 | 18735 |
| Tsang, 2005  [69] | C | China | Regional | Healthy subjects from 22 hospitals and clinics of the Hospital Authority in Hong Kong. | Jamar hydraulic | 21 - 70 | 544 |
| Tveter, 2014  [70] | C | Norway | Local | Volunteers recruited from a range of work sites, schools, community centres for older adults. | Baseline | 18 - 90 | 370 |
| Vianna, 2007  [71] | C | Brazil | Facility | Those attending a private exercise medicine clinic. | Takei Digital Grip Dynamometer | 18 - 75 | 2477 |
| Wang, 2010  [72] | NS | Taiwan, Province of China | NS | Volunteers but source(s) NS. | Jamar hydraulic | 60 - 89 | 176 |
| Werle, 2009  [73] | C | Switzerland | Local | Shopping centres and malls, secondary schools, senior sports groups and senior residences. | Jamar hydraulic | 18 - 84 | 922 |
| Wu, 2009  [74] | C | Taiwan, Province of China | National | The research team visited universities, mountain villages, public parks, markets, community halls, churches and temples. Access to volunteers was gained through community gatekeepers, district nurses, priests, and local community leaders. | Jamar hydraulic | 20 - 74 | 435 |
| Yim, 2003  [75] | C | Korea, Republic of | Facility | Students in an elementary school in Suwon city, Korea | Jamar hydraulic | 7 - 12 | 712 |
| Yoshimura, 2011  [76] | Sf | Japan | National | Second wave of a large-scale population cohort study: the ROAD study (research on osteoarthritis / osteopororis against disability) | Toei Light handgrip dynamometer | 40 - 79 | 1776 |
